# Multi-omics analysis reveals neuroinflammation, activated glial signaling, and dysregulated synaptic signaling and metabolism in the hippocampus of aged mice

**DOI:** 10.3389/fnagi.2022.964429

**Published:** 2022-11-03

**Authors:** Yinzhong Lu, Kejia Xu, Dongyang Lin, Shuyan Wang, Rao Fu, Xiaobei Deng, Giorgia Croppi, Junjie Zhang

**Affiliations:** ^1^Department of Anesthesiology and Hongqiao International Institute of Medicine, Tongren Hospital, Shanghai Jiao Tong University School of Medicine, Shanghai, China; ^2^Department of Neurology, Tongren Hospital, Shanghai Jiao Tong University School of Medicine, Shanghai, China; ^3^Faculty of Public Health, Shanghai Jiao Tong University School of Medicine, Shanghai, China; ^4^Connect Biopharma Ltd., Taicang, China

**Keywords:** brain ageing, neuroinflammation, microbiota-derived metabolite, learning and memory, synaptic plasticity, transcriptomics, metabolomics, multi-omics analysis

## Abstract

Aging is an intricate biological event that occurs in both vertebrates and invertebrates. During the aging process, the brain, a vulnerable organ, undergoes structural and functional alterations, resulting in behavioral changes. The hippocampus has long been known to be critically associated with cognitive impairment, dementia, and Alzheimer’s disease during aging; however, the underlying mechanisms remain largely unknown. In this study, we hypothesized that altered metabolic and gene expression profiles promote the aging process in the hippocampus. Behavioral tests showed that exploration, locomotion, learning, and memory activities were reduced in aged mice. Metabolomics analysis identified 69 differentially abundant metabolites and showed that the abundance of amino acids, lipids, and microbiota-derived metabolites (MDMs) was significantly altered in hippocampal tissue of aged animals. Furthermore, transcriptomic analysis identified 376 differentially expressed genes in the aged hippocampus. A total of 35 differentially abundant metabolites and 119 differentially expressed genes, constituting the top 200 correlations, were employed for the co-expression network. The multi-omics analysis showed that pathways related to inflammation, microglial activation, synapse, cell death, cellular/tissue homeostasis, and metabolism were dysregulated in the aging hippocampus. Our data revealed that metabolic perturbations and gene expression alterations in the aged hippocampus were possibly linked to their behavioral changes in aged mice; we also provide evidence that altered MDMs might mediate the interaction between gut and brain during the aging process.

## Introduction

Aging is an intricate process involving progressive functional degeneration at both the cellular and organ levels. Multiple factors, including nutrition, metabolism, and gut microbiota, can influence the aging process (O’Toole and Jeffery, 2015; DeJong et al., 2020; Hamrick and Stranahan, 2020). In addition, these factors are highly associated with genetic loci, altered gene expression, and gene regulatory networks (Hawrylycz et al., 2012). Accordingly, aging is a predominant risk factor for many common medical conditions, including diabetes, Alzheimer’s disease (AD), stroke, and chronic obstructive pulmonary disease (Childs et al., 2015). However, how the aging process contributes to the onset and progression of these diseases is still poorly understood.

A healthy aging brain is vital to life and longevity. Studies have shown that aging contributes to unique structural and anatomical changes in the brain, alterations in network connectivity and synaptic plasticity, cognitive decline, neuroinflammation, and dysregulation of metabolism, which can lead to the development of neurological diseases (Colantuoni et al., 2011; Cribbs et al., 2012; Liang et al., 2017; Mattson and Arumugam, 2018; Hamrick and Stranahan, 2020). The hippocampus is the primary brain region responsible for cognition and its impairment during the aging process can lead to AD and other neurological disorders (Small et al., 2011; Hou et al., 2019). Single omics techniques have been widely used to explore the alterations occurring in brain aging from different levels (Hawrylycz et al., 2012; Hamezah et al., 2018; Adav and Wang, 2021), including the transcriptome, epigenome, proteome, metabolome, gut microbiome, and so on. In the aging hippocampus, the alterations of genes, metabolites, and gut microbiome have been explored by single omics analysis. These findings showed that genes/proteins related to neuroinflammation and synaptic signaling (Xu et al., 2007; Youm et al., 2013; Stilling et al., 2014; Mangold et al., 2017; Miller et al., 2017; Hamezah et al., 2018; Gonzalez-Velasco et al., 2020; Peng et al., 2021), and metabolites categorized in the metabolism of amino acids, lipids, glucose and energy expenditure (Liu et al., 2009; Paban et al., 2010; Lin et al., 2016; Durani et al., 2017; Ding et al., 2021; Ge et al., 2021; Vallianatou et al., 2021) were altered in rodents and humans. In addition, several defined microbiota-derived metabolites (MDMs) changed in the aged hippocampus (Noack et al., 2000; Wang et al., 2011; Bennett et al., 2013) and they were shown to possibly affect hippocampus or brain functions, such as learning, memory and synaptic plasticity (Li et al., 2018; Madeo et al., 2018; Govindarajulu et al., 2020; Schroeder et al., 2021). However, there is a lack of comprehensive integrative information from multiple levels during hippocampus aging. Therefore, more comprehensive integrative studies of the aging hippocampus, particularly from a global and holistic perspective, are needed to confirm these possibilities, which might provide more possible strategies for anti-aging.

Multi-omics analyses exhibit the advantages of allowing a more detailed understanding of disease pathogenesis from multiple perspectives (Rivero-Segura et al., 2020), and it was widely applied to brain aging and neurodegenerative studies (Mahajan et al., 2020; Rivero-Segura et al., 2020). However, no multi-omics analyses were documented in the aged hippocampus. Here, based on multi-omics analysis, we aim to examine the hippocampus-related behavioral changes of aged mice and explore how their changes linked to their metabolic and gene expression profiles change in the aged hippocampus to better understand the molecular mechanisms underlying hippocampal aging from multiple spheres in this study. Hippocampus-related behavioral tests showed that aged mice exhibited anxiety-like behavior, impaired associative learning and memory, and decreased locomotor activity. Multi-omics (metabolomics and transcriptomics) analysis revealed the presence of neuroinflammation, activated glial signaling, dysregulated synaptic signaling, and impaired metabolism in the hippocampus of aged mice. The data further showed that MDMs were differentially expressed in the hippocampi of aged animals.

## Materials and methods

### Materials

All chemicals and solvents were of analytical or HPLC grade. Acetonitrile, methanol, ammonium hydroxide, and ammonium acetate were purchased from CNW Technologies GmbH (Düsseldorf, Germany). L-2-chlorophenylalanine was obtained from Shanghai Hengchuang Biotechnology Co., Ltd. (Shanghai, China).

### Mice

The young and middle-aged male C57/BL6J mice used in this study were purchased from a specific-pathogen-free (SPF) facility in the Shanghai Model Organism Center and were maintained in a SPF animal hood with free access to food and water. All procedures were carried out according to the animal experimentation regulations and approved by the ethics committee of Tongren Hospital, Shanghai Jiao Tong University School of Medicine.

### Open field test

The open field test (OFT) is widely employed to examine rodent locomotor activity and exploratory behaviors. Based on our previous study (Xu et al., 2022), all open field testing was performed inside an arena (50 cm long × 50 cm wide × 40 cm high) that was divided into a central and peripheral regions using the VisuTrack system (Shanghai XinRuan Information Technology Co., Ltd., Shanghai, China). Animals were removed from their home cage by the tail and placed directly into the center of the open field. Tracking/recording was initiated upon the first break of the locomotion grid beam and lasted for 5 min and the trajectory of the mice was analyzed using the VisuTrack system. The total distance traveled was recorded to evaluate the movement ability of the mice. The number of entries into and the time spent in the central region by each animal were measured to detect the levels of anxiety.

### Shuttle box test

The shuttle box test is typically employed to examine learning and memory abilities in rodents. The avoidance response was assessed as previously described (Ortiz et al., 2010), with a small modification. Briefly, mice were tested in an automatic, four-channel, two-way shuttle box system (Ugo Basile, Italy). Each animal received one training session per day for 7 days and was then tested once on days 10 and 25, respectively. The training program consisted of a 3-min adaptation period followed by 30 trials with an intertrial interval of 20 ± 5 s. In each trial, a tone (2,400 Hz, 25% intensity) and white light were simultaneously presented for 10 s as the conditioned stimulus (CS). After 5 s, a 0.2-mA electric shock (the unconditioned stimulus [US]) was delivered for a maximum of 10 s. An avoidance response was defined as the animal crossing to the opposite compartment of the box after the start of the CS but before the US was delivered. An escape response was defined as the crossing occurring when the floor shock was being delivered. Response latencies were determined as the time (s) from the onset of the CS until the animal crossed into the opposite compartment. The number of crossings (n) during the intertrial interval (ITI) served as a measure of general activity. The apparatus was cleaned with water between animals.

### Tissue preparation

Following anesthesia with 10% chloral hydrate, whole blood was collected from each mouse *via* the eyeball and the animals were euthanized by cervical dislocation. The hippocampus was then freshly dissected and snap-frozen in liquid nitrogen for RNA isolation and metabolite extraction.

### Liquid chromatography–mass spectrometry-based metabolomics analysis

Metabolites were extracted from ∼15 mg of snap-frozen hippocampal tissue using a mixture of acetonitrile: methanol: water in a ratio of 2:2:1 (mixed with the internal standard) as previously described (Want, 2018). The supernatant was used for liquid chromatography-mass spectrometry (LC-MS) analysis. Ultra-high performance liquid chromatography (UHPLC) separation was performed using a 1290 UHPLC System (Agilent Technologies, Santa Clara, CA, USA) equipped with a UPLC BEH Amide column (2.1 × 100 m, 1.7 μm, Waters, USA). A Q-Exactive Orbitrap mass spectrometer (Thermo Fisher Scientific, San Jose, CA, USA) was employed to acquire a full scan of MS/MS spectra in information-dependent acquisition (IDA) mode under the control of Xcalibur acquisition software (v. 4.0.27; Thermo Fisher Scientific). MS was performed at AigenX Biosciences Co., Ltd. (Shanghai, China).

The acquired MS/MS spectra were processed as previously described (Smith et al., 2006). Briefly, the raw data were converted to the mzXML format using ProteoWizard and processed for peak detection, extraction, alignment, and integration based on XCMS. Then, an in-house MS2 database (BTDB) was applied to metabolite annotation with the cutoff set at 0.3. The final dataset, which included information relating to sample name, peak number, and normalized peak area, was imported into SIMCA15.0.2 (Sartorius Stedim Data Analytics AB, Umea, Sweden) for multivariate analysis. The data were analyzed by principal component analysis (PCA) to visualize the distribution and grouping of the samples. A 95% confidence interval in the PCA score plot was used as the threshold for identifying potential outliers in the dataset. To visualize group segmentation and identify significantly altered metabolites, supervised orthogonal partial least-squares discriminant analysis (OPLS-DA) was applied to separate the aged group from the young group, followed by a 7-fold cross-validation test and permutation tests (200 permutations) to validate the OPLS-DA model. The variable importance in the projection (VIP) value was also obtained by OPLS-DA analysis. Metabolites with a VIP score > 1 (OPLS-DA test) and a *p*-value < 0.05 (Student’s *t*-test) were considered to be significantly differential metabolites (DMs). In addition, the Kyoto Encyclopedia of Genes and Genomes (KEGG)^1^ and OEcloud^2^ were used for pathway enrichment analysis.

### Library preparation and RNA-seq analysis

Total RNA was extracted from the hippocampus and purified for RNA-seq library preparation as previously described (Lu et al., 2017, 2021). The sequencing libraries were prepared using the TruSeq Stranded Total RNA Library Prep Kit (Illumina, San Diego, CA, USA) according to the manufacturer’s instructions. Briefly, the mRNA was purified and fragmented, followed by first-and then second-strand cDNA synthesis before digestion with polymerase I and RNase H. The remaining overhangs were blunted *via* exonuclease/polymerase activities and purified. After adenylation of the 3’ ends of the DNA fragments, Illumina PE adapter oligonucleotides were ligated to prepare for hybridization. The library fragments were purified using the AMPure XP System (Beckman Coulter, Beverly, CA, USA) and selectively enriched using an Illumina PCR Primer Cocktail in a 15-cycle PCR. Products were purified (AMPure XP system) and quantified using a high-sensitivity DNA assay on a Bioanalyzer 2100 System (Agilent). The prepared libraries were sequenced by AigenX Bioscience Co., Ltd. using a HiSeq 2500 platform (Illumina), yielding paired-end (2 × 125 bp) reads. All the samples from each group were sequenced in biological triplicates.

The original raw data in FASTQ format generated by the HiSeq 2500 platform was further filtered using Cutadapt (v.1.15) software to obtain clean data for subsequent analysis. The clean data were then mapped to the mouse reference genome (GRCm38.p2)^3^ using HISAT2 software.^4^ HTSeq (v.0.9.1) statistics were used to compare the read count with the original expression. Gene expression was normalized based on FPKM values. DESeq (1.30.0) was employed to identify differentially expressed genes using | log2FoldChange| > 1 and a *p*-value < 0.05 as the threshold. Subsequently, gene ontology (GO) or KEGG pathway enrichment analysis of the DEGs was undertaken using the OECloud tool.^5^ The RNA-seq data have been deposited in the NCBI BioProject database^6^ under accession number PRJNA PRJNA842200.

### Reverse transcription and qPCR

RNA isolation, reverse transcription, and qPCR were performed as previously described (Lu et al., 2017, 2021). Briefly, RNA was isolated with TRIzol reagent and reverse transcribed (1 μg) using the ReverTra Ace Kit (TOYOBO, Nipro, Osaka, Japan). The resulting cDNA was used as a template for qPCR which was performed in a LightCycler 480 system (Roche) using a 2 × Power SYBR Green Mix (Applied Biosystems, Carlsbad, CA, USA). Gene expression levels were normalized to that of *GAPDH* and calculated using the 2^–ΔΔ*Ct*^ method. The sequences of the primer used for qPCR are available on request from the corresponding authors.

### Integrative transcriptomic and metabolomic analysis

The R package was used to calculate Pearson’s correlation coefficients between transcriptomic and metabolomic data based on DEG and differentially abundant metabolite (DM) data, as previously described (Luo and Brouwer, 2013). The DEGs and DMs were mapped to the KEGG database and a correlation-based (|r| ≥ 0.9825 and *p* < 0.00046 [top 200]) gene–metabolite co-expression network was built and visualized using Cytoscape (v.3.5.1) (Gao et al., 2010).

### Statistical analysis

Data were analyzed in GraphPad Prism 8 and are shown as means ± SEM. The raw data for each group were analyzed by ANOVA with a *post-hoc* test or Student’s *t*-test as indicated in the figure legends. *p*-values < 0.05 were considered significant.

## Results

### Anxiety-like behavior and locomotor activity were altered in middle-aged mice

**FIGURE 1:**
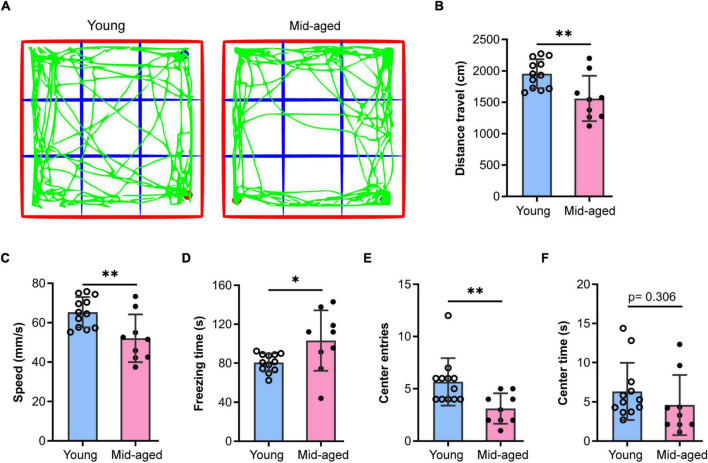
Open field test to study the behaviors of young and middle-aged mice. **(A)** Representative trajectory of young or middle-aged (Mid-aged) male mice in the open-field test. **(B–F)** Statistic data of the open field test parameters. Data shown are means with SEM. The distance travel **(B)**, speed **(C)**, freezing time **(D)**, center entries **(E)**, and center time **(F)** were quantified by the Open field test during 5-min monitoring. Student’s *t*-test and the *p*-value were shown in each bar chart, *p < 0.05, **p < 0.01; the hollow dot and the solid circle show the sampling dataset collected from the young group (*n* = 12) and the middle-aged group (*n* = 9, Mid-aged).

Middle-aged (12∼14 months old) and aged mice (older than 18 months) are commonly used for studies on aging (Shoji et al., 2016; Boehme et al., 2020; Cizeron et al., 2020; Ding et al., 2021; Schroeder et al., 2021; Mossad et al., 2022). Here, we used 13∼14 month-old and 22–23-month-old animals as middle-aged and aged mice, respectively. Additionally, young (2–3 months old) mice were used to characterize some of the typical mechanisms that fail during old age. Given that alterations in anxiety levels impact cognitive performance, we first examined exploratory behavior, anxiety-like behavior, and locomotor activity in middle-aged mice using the OFT. The results showed that the middle-aged mice (*n* = 9) traveled shorter distances (*p* < 0.01), were slower (*p* < 0.01), and spent substantially more time frozen (*p* < 0.05) than the young mice (*n* = 12) (Figures 1A–D), indicating that locomotor activity was decreased in the former. Additionally, compared to the young group, middle-aged mice made fewer entries into (*p* < 0.01, Figure 1E) and spent less time in the central region (*p* = 0.306, Figure 1F), which suggested that middle-aged mice exhibit anxiety-like behavior, consistent with the results of previous studies (Shoji et al., 2016).

### Associative learning and memory were impaired in aged mice

**FIGURE 2:**
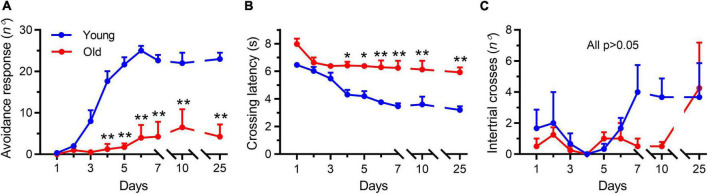
Aging impaired the active avoidance performance in male mice. Data shown are means with SEM. **(A)** Progression of active avoidance responses during the training phase. Aged mice (Old) did not increase the number of avoidance responses during the training phase (**p < 0.001). **(B)** Time course of crossing latencies for aged mice (Old) during the training phase. Old mice did not decrease escape latency during training (*p < 0.01, **p < 0.001). **(C)** Number of intertrial crosses. From day 1, there was no significant difference between young and aged mice (Old) in the number of intertrial crosses. Statistics were performed with repeated-measures two-way ANOVA, followed by post-Bonferroni’s test, *n* = 3–4 per group.

In the shuttle box test, the young mice learned the avoidance response within the first 4 days of training, whereas the aged mice (22–23 months) were unable to learn it throughout the training period (Figure 2). Differences between the two groups were significant on the fourth training day and persisted throughout the experiment (Figure 2A, *p* < 0.001), demonstrating that avoidance learning was impaired in aged mice. In addition, the latency to crossing of young mice was continuously reduced compared with that in the aged mice (Figure 2B), which was again indicative of impaired learning in the latter group. Meanwhile, the number of random intertrial crosses showed no noticeable difference at the beginning of training. However, both on the training (day 7) and testing days (day 10), the number of random intertrial crosses decreased in the group of aged mice compared with that seen in the group of young animals (*p* = 0.40 and *p* = 0.61, respectively, Figure 2C).

Interestingly, after 2 weeks (testing day 25), the tendency to decrease locomotion disappeared in the aged mice (Figure 2C, *p* > 0.99), but the young mice continued to keep their good testing records (Figures 2A,B), indicating that a good memory was retained in the young mice but not in the aged animals. These results showed a tendency to decrease the locomotor activity in the aged mice during the persistent training days, which further examined the reduced locomotor activity by OFT experiments. Collectively, these results demonstrated that the aged mice exhibited degeneration-associated learning and memory impairment and decreased locomotor activity, consistent with previous studies that employed different methods for evaluating learning and memory ability (Schroeder et al., 2021).

### Metabolomic profiling of the aging hippocampus

**FIGURE 3:**
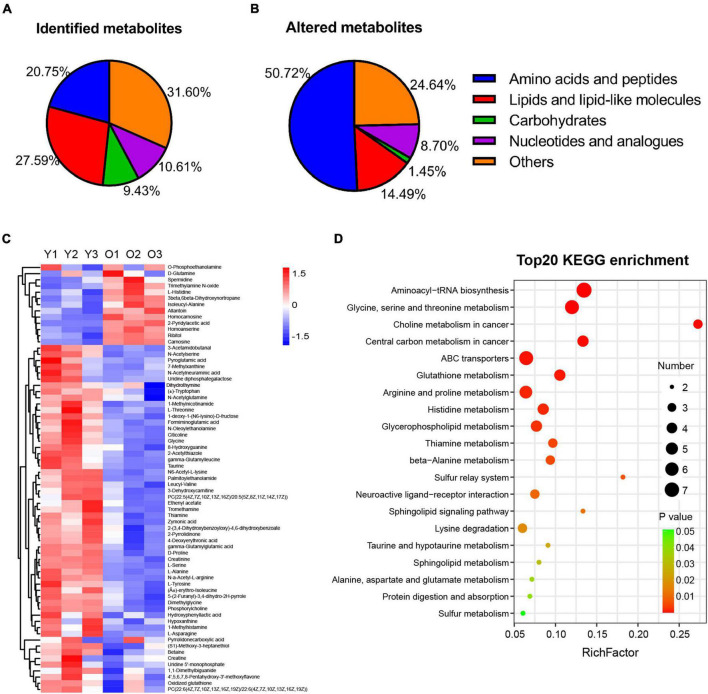
Metabolic profiling and altered metabolism-related pathways of hippocampus tissue in male aged mice. **(A,B)** Pie graph of the metabolites class composition of identified and significantly altered metabolites in the hippocampus of male aged mice. **(C)** Hierarchical heatmap analysis of the relative content of DEMs in hippocampus from male aged (O1–O3, *n* = 3) and young mice (Y1–Y3, *n* = 3). **(D)** Bubble illustration of top20 ranked enriched KEGG pathway terms. The diameter of the solid circle denotes the number of DEMs enriched and the color showing the *p*-value in the corresponding pathway.

Numerous studies have reported the changes occurring in the metabolome of tissues or organs during the aging process (Mattson and Arumugam, 2018; Dall and Færgeman, 2019; Hamrick and Stranahan, 2020; Petr et al., 2021). Here, we investigated the changes occurring in the hippocampal metabolome of aged mice during the aging process and compared them with those of young mice. We undertook LC–MS-based metabolomic profiling of hippocampal tissues from aged (22–23 months old) and young mice (*n* = 3 per group) and identified a total of 424 metabolites, 262 in positive ion mode and 162 in negative ion mode (Figure 3A and Supplementary Table 1). Furthermore, the OPLS-DA score plot for metabolites detected in both modes clearly discriminated between the young and aged groups (Supplementary Figure 1).

Furthermore, among the 424 metabolites identified, 69 were found to be differentially abundant (VIP > 1, *p* < 0.05) in the aged hippocampus compared with the young hippocampus, 13 of which were upregulated and 56 downregulated (Table 1). The DMs were classified into several subclasses, including amino acids and peptides, lipids, carbohydrates, and nucleotides. Among them, amino acids and peptides (*n* = 35) accounted for the greatest proportion of the significantly altered metabolites in the aged hippocampus (Figure 3B). The identified DMs are shown in Table 1 and Figure 3C. Notably, almost all the altered amino acid and lipid metabolites in the aged hippocampus were downregulated. Upregulated DMs included trimethylamine N-oxide (TMAO), allantoin, spermidine, carnosine, homocarnosine, and homoanserine. KEGG pathway enrichment analysis of the DMs showed that amino acid metabolism- and lipid metabolism-related pathways were significantly altered in the aged hippocampus (Figure 3D). Combined, these results suggested that amino acid and lipid homeostasis were disrupted in the aging mouse hippocampus, which further documented the previous reports or reviews (Liu et al., 2009; Paban et al., 2010; Lin et al., 2016; Durani et al., 2017; Adav and Wang, 2021; Ding et al., 2021; Ge et al., 2021; Vallianatou et al., 2021).

**TABLE 1:** The altered differential metabolites in the aged hippocampus.

No	Metabolites	HMDB	KEGG	VIP	*P*-value	FC	Class
1	(±)-erythro-Isoleucine	HMDB0033923		1.81	0.0174	0.58	Amino acids and peptides
2	(±)-Tryptophan	HMDB30396		1.69	0.0349	0.80	Amino acids and peptides
3	(S1)-Methoxy-3-heptanethiol	HMDB0032380		1.69	0.0339	0.83	Amino acids and peptides
4	1,1-Dimethylbiguanide	HMDB0001921	C07151	1.60	0.0478	0.59	Amino acids and peptides
5	1-deoxy-1-(N6-lysino)-D-fructose	HMDB0062186		1.98	0.0016	0.67	Amino acids and peptides
6	2-Pyridylacetic acid	HMDB0060722		2.02	0.0074	2.57	Amino acids and peptides
7	Betaine	HMDB0000043	C00719	1.77	0.0162	0.69	Amino acids and peptides
8	Carnosine	HMDB0000033	C00386	2.02	0.0001	1.67	Amino acids and peptides
9	Creatine	HMDB0000064	C00300	1.65	0.0451	0.86	Amino acids and peptides
10	D-Glutamine	HMDB0003423	C00819	1.72	0.0285	1.08	Amino acids and peptides
11	Dimethylglycine	HMDB0000092	C01026	1.90	0.0059	0.78	Amino acids and peptides
12	D-Proline	HMDB0003411	C00763	1.92	0.0029	0.77	Amino acids and peptides
13	Formiminoglutamic acid	HMDB0000854	C00439	1.81	0.0260	0.58	Amino acids and peptides
14	Gamma-Glutamyl glutamic acid	HMDB0011737	C05282	1.83	0.0030	0.46	Amino acids and peptides
15	Gamma-Glutamyl leucine	HMDB0011171		1.76	0.0258	0.79	Amino acids and peptides
16	Glycine	HMDB0000123	C00037	1.91	0.0071	0.70	Amino acids and peptides
17	Homoanserine	HMDB0005767		1.96	0.0008	1.94	Amino acids and peptides
18	Homocarnosine	HMDB0000745	C00884	1.99	0.0019	1.63	Amino acids and peptides
19	Hydroxyphenyl lactic acid	HMDB0000755	C03672	1.82	0.0215	0.75	Amino acids and peptides
20	Isoleucyl-Alanine	HMDB0028900		1.73	0.0298	1.30	Amino acids and peptides
21	L-Alanine	HMDB0000161	C00041	1.96	0.0022	0.79	Amino acids and peptides
22	L-Asparagine	HMDB0000168	C00152	1.79	0.0328	0.76	Amino acids and peptides
23	Leucyl-Valine	HMDB0028942		1.89	0.0056	0.72	Amino acids and peptides
24	L-Histidine	HMDB0000177	C00135	1.70	0.0323	1.25	Amino acids and peptides
25	L-Serine	HMDB0000187	C00065	2.01	0.0001	0.60	Amino acids and peptides
26	L-Threonine	HMDB0000167	C00188	1.94	0.0054	0.67	Amino acids and peptides
27	L-Tyrosine	HMDB0000158	C00082	1.93	0.0044	0.65	Amino acids and peptides
28	N6-Acetyl-L-lysine	HMDB0000206	C02727	1.96	0.0012	0.56	Amino acids and peptides
29	N-a-Acetyl-L-arginine	HMDB0004620		1.99	0.0008	0.63	Amino acids and peptides
30	N-Acetylglutamine	HMDB0006029		1.80	0.0209	0.77	Amino acids and peptides
31	N-Acetylneuraminic acid	HMDB0000230	C19910	1.79	0.0185	0.91	Amino acids and peptides
32	N-Acetylserine	HMDB0002931		1.98	0.0008	0.68	Amino acids and peptides
33	Oxidized glutathione	HMDB0003337	C00127	1.72	0.0269	0.81	Amino acids and peptides
34	Pyroglutamic acid	HMDB0000267	C01879	1.70	0.0397	0.82	Amino acids and peptides
35	Taurine	HMDB0000251	C00245	1.82	0.0150	0.83	Amino acids and peptides
36	Ribitol	HMDB0000508	C00474	1.97	0.0022	1.15	Carbohydrates
37	3-Dehydroxycarnitine	HMDB0006831	C05543	1.79	0.0176	0.79	Lipid and lipid-like molecules
38	4-Deoxyerythronic acid	HMDB0000498		1.74	0.0203	0.76	Lipid and lipid-like molecules
39	Citicoline	HMDB0001413	C00307	1.85	0.0122	0.82	Lipid and lipid-like molecules
40	Ethenyl acetate	HMDB0031209	C19309	1.68	0.0436	0.82	Lipid and lipid-like molecules
41	N-Oleoylethanolamine	HMDB0002088		1.88	0.0136	0.56	Lipid and lipid-like molecules
42	O-Phosphoethanolamine	HMDB0000224	C00346	1.75	0.0348	0.86	Lipid and lipid-like molecules
43	Palmitoylethanolamide	HMDB0002100	C16512	1.96	0.0023	0.66	Lipid and lipid-like molecules
44	PC (22:5/20:5)	HMDB0008675	C00157	1.73	0.0317	0.45	Lipid and lipid-like molecules
45	PC (22:6/22:6)	HMDB0008748	C00157	1.62	0.0482	0.82	Lipid and lipid-like molecules
46	Phosphorylcholine	HMDB0001565	C00588	1.93	0.0024	0.69	Lipid and lipid-like molecules
47	7-Methylxanthine	HMDB0001991	C16353	1.72	0.0302	0.83	Nucleotides and analogs
48	8-Hydroxyguanine	HMDB0002032	C20155	1.68	0.0404	0.83	Nucleotides and analogs
49	Dihydrothymine	HMDB0000079	C00906	1.68	0.0355	0.86	Nucleotides and analogs
50	Hypoxanthine	HMDB0000157	C00262	1.58	0.0455	0.44	Nucleotides and analogs
51	Uridine 5′-monophosphate	HMDB0000288	C00105	1.68	0.0459	0.77	Nucleotides and analogs
52	Uridine diphosphategalactose	HMDB0000302	C00052	1.72	0.0315	0.86	Nucleotides and analogs
53	1-Methylhistamine	HMDB0000898	C05127	1.86	0.0126	0.74	Others
54	1-Methylnicotinamide	HMDB0000699	C02918	1.91	0.0084	0.49	Others
55	2-(3,4-Dihydroxybenzoyloxy)-4,6-dihydroxybenzoate	HMDB0059651	C04524	1.59	0.0445	0.63	Others
56	2-Acetylthiazole	HMDB0032964		1.70	0.0358	0.85	Others
57	2-Pyrrolidinone	HMDB0002039	C11118	1.71	0.0267	0.77	Others
58	3-Acetamidobutanal	HMDB0059649		1.95	0.0064	0.41	Others
59	3beta,6beta-Dihydroxynortropane	HMDB0038949		1.76	0.0248	1.11	Others
60	4’,5,6,7,8-Pentahydroxy-3’-methoxyflavone	HMDB0033648		1.82	0.0168	0.88	Others
61	5-(2-Furanyl)-3,4-dihydro-2H-pyrrole	HMDB0040013		1.10	0.0283	0.33	Others
62	Allantoin	HMDB0000462	C01551	1.86	0.0148	1.55	Others
63	Creatinine	HMDB0000562	C00791	1.99	0.0061	0.75	Others
64	Pyrrolidonecarboxylic acid	HMDB0000805	C02237	1.80	0.0186	0.91	Others
65	Spermidine	HMDB0001257	C00315	1.68	0.0438	1.17	Others
66	Thiamine	HMDB0000235	C00378	1.76	0.0202	0.75	Others
67	Trimethylamine N-oxide	HMDB0000925	C01104	1.91	0.0051	2.15	Others
68	Tromethamine	HMDB0240288	C07182	1.86	0.0098	0.83	Others
69	Zymonic acid	HMDB0031210		1.77	0.0238	0.70	Others

VIP, variable importance in the projection; FC, foldchange, indicating the relative metabolite abundance of aged to young. PC (22:5/20:5), PC [22:5(4Z,7Z,10Z,13Z,16Z)/20:5(5Z,8Z,11Z,14Z,17Z)]; PC (22:6/22:6), PC [22:6(4Z,7Z,10Z,13Z,16Z,19Z)/22:6(4Z,7Z,10Z,13Z,16Z,19Z)].

### The expression of gut microbiota-derived metabolites was altered in the aging hippocampus

**FIGURE 4:**
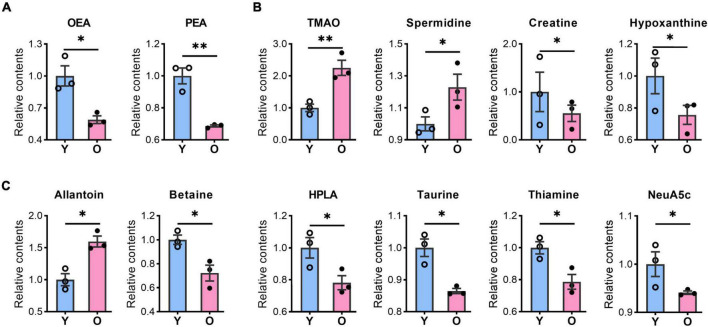
The contents of bioactive lipids and microbiota-derived metabolites were changed in the aged hippocampus of male mice. The relative contents of bioactive lipids, OEA, and PEA **(A)**; microbiota-derived metabolites, TMAO, spermidine, creatine, and hypoxanthine **(B)**, allantoin, betaine, HPLA, taurine, thiamine, and Neu5Ac **(C)** were altered in the aged (O) and young (Y) hippocampus. Data are calculated as the relative contents of the young group and represent the means ± SEM. Student’s *t*-test, *n* = 3 per group, *p < 0.05, **p < 0.01). OEA, N-oleoylethanolamine; PEA, palmitoylethanolamide; TMAO, trimethylamine N-oxide; HPLA, hydroxyphenyl lactic acid; Neu5Ac, N-acetylneuraminic acid.

Our metabolomic analysis identified many known MDMs, including short-chain fatty acids, indoles, phenols, nucleotides, and amino acids. Intriguingly, the abundance of several MDMs, such as TMAO and spermidine (Noack et al., 2000; Wang et al., 2011; Bennett et al., 2013), was significantly changed in the hippocampus of aging mice (Figure 4 and Supplementary Table 2), which possibly taking up ∼33.3% (23/69) of identified DMs. TMAO, derived from trimethylamine, has been reported to accelerate the brain aging process by impairing cognition and decreasing synaptic plasticity (Li et al., 2018; Govindarajulu et al., 2020). Here, we found that the TMAO content was significantly enriched in the aged hippocampus (Figure 4B). Meanwhile, spermidine that was slightly upregulated in aging mice, is thought to exert protective effects on brain aging, and its dietary intake improves brain behaviors (Madeo et al., 2018; Schroeder et al., 2021). Additionally, hypoxanthine exhibits neurotoxic effects (Biasibetti et al., 2017; Biasibetti-Brendler et al., 2018) was decreased in aged group.

Among other potential MDMs that play a protective role in the brain or neurons, the levels of betaine, creatine, thiamine, Neu5Ac, and taurine levels were significantly decreased, whereas that of allantoin was increased, in the hippocampus of aged mice (Figures 4B,C). Meanwhile, amino acids such as dimethylglycine, glycine, L-alanine, L-asparagine, L-serine, L-tyrosine, (±)-Tryptophan, L-threonine, pyroglutamic acid, and L-histidine (Figure 3C), also possibly derived from the gut microbiota (Zheng et al., 2011; Zhao et al., 2017), were all upregulated in aged animals. These results suggested that the abundance of many potential MDMs was likely to be altered, which likely negatively affected the hippocampus during the aging process.

### Transcriptomic analysis of the aged hippocampus

**FIGURE 5:**
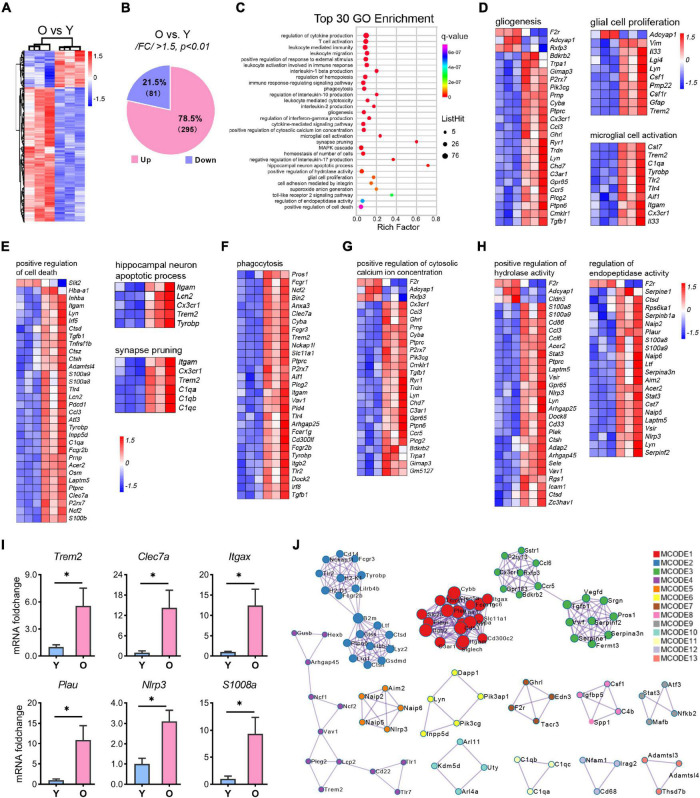
RNA-seq analysis of the differential expressional genes enriched in aging. **(A,B)** Hierarchical heatmap analysis [**(A)**, *n* = 3 per group] and pie graph of the up-and down-regulated genes composition **(B)** of the differentially expressed genes (DEGs) as determined by the selection criteria: | FC| > 1.5 and *p* < 0.01. **(C)** Top 30 GO terms enriched by the MetaScape tool. The diameter of the solid circle denotes the number of DEGs enriched and the color showing the *p*-value in the corresponding pathway. **(D–H)** Gene cluster enriched in gliogenesis, glia cell proliferation, and microglia activation **(D)**; positive regulation of cell death, hippocampus neuron death, and synapse pruning **(E)**; phagocytosis **(F)**; positive regulation of cytosolic calcium ion concentration **(G)**; and regulation of endopeptidase activity and positive regulation of hydrolase activity **(H)**. **(I)** qRT-PCR analysis of selected genes related to microglial activation and neuroinflammation. Data are calculated as the mRNA foldchange of the young group and represent the means ± SEM. Unpaired Student’s *t*-test, *n* = 3–6 per group *p < 0.05. **(J)** The Hub genes clustering by the protein-protein interaction network as determined by the MCODE tool of MetaScape.

Next, we performed a transcriptomic analysis to elucidate how changes in behavior and metabolomics are related to gene expression during hippocampal aging. In total, 39, 457, 406 ∼49, 075, 794 raw reads were sequenced and uniquely mapped to the mouse genome. We compared the hippocampal transcriptome signatures of aged and young mice, with the PCA analysis showing clear segregation between the aged and young animals (Supplementary Figure 2A). Moreover, we undertook a DESeq analysis to screen the differentially expressed genes (DEGs) using | Log2FC| > 0.58 and *p* < 0.01 as cutoffs and identified 295 genes that were upregulated and 81 that were downregulated in the aged hippocampus (Figures 5A,B, Supplementary Figure 2B and Supplementary Table 3).

### The gene regulatory network was altered in the aged hippocampus

GO enrichment analysis of 376 DEGs using the online tool MetaScape showed that they clustered into 30 significant top-ranked GO terms (*p* < 7.1E-07, Figure 5C). Notably, the DEGs were primarily associated with inflammation-related pathways, including regulation of cytokine production, leukocyte-mediated immunity, interleukin-1β (IL-1β) production, immune response-regulating signaling pathway, cytokine-mediated signaling pathway, and Toll-like receptor 2 signaling pathway (Figure 5C and Supplementary Figure 3). Moreover, brain function-related terms such as glial function (microglial activation and gliosis; Figure 5D), neuron death, synapse pruning (Figure 5E), tissue homeostasis (cytosolic calcium ion, hydrolase activity, endopeptidase activity, phagocytosis, cell death, and superoxide ion generation; Figures 5F–H and Supplementary Figure 3), and MAPK cascade were overrepresented in the aged group (Supplementary Figure 3). To validate these signaling pathways, we selected several genes related to microglial activation and neuroinflammation and submitted them to qPCR analysis. The results were consistent with those of the RNA-seq analysis (Figure 5I and Supplementary Table 3).

Based on GO terms, protein-protein interaction (PPI) network analysis through MCODE identified 13 clusters (Figure 5J). The top 3 clusters included the GO: 0006954 (inflammatory response, *p* = E-32.1), GO: 0001817 (regulation of cytokine production, *p* = E-27.6), and GO: 0002443 (leukocyte mediated immunity, *p* = E-21.6) categories, thus identifying a combinatorial regulatory network active in inflammation-related events in the nervous system during the aging process. In addition, apart from the critical nodes, analysis using MCODE identified other hub gene clusters, as shown in Figure 5J. These enriched GO terms and PPI network pinpointed the inflammation-related pathways in the aging hippocampus, which corroborated previous studies (Cribbs et al., 2012; Youm et al., 2013; Stilling et al., 2014; Di Benedetto et al., 2017; Mangold et al., 2017).

### Integrated pathway and network analysis

**FIGURE 6:**
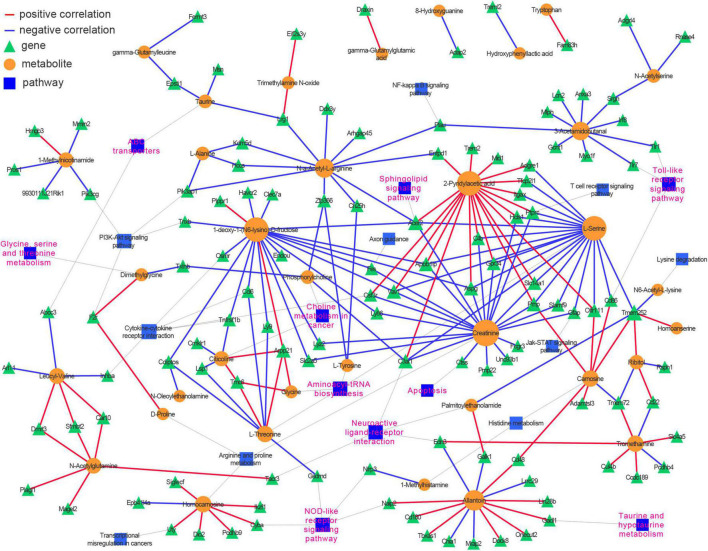
Integrative analysis of transcriptomics and metabolomics identifies neuroinflammation, amino acids metabolism, sphingolipid signaling pathways, and neuroactive ligand and receptor interaction over presented in the hippocampus of aged mice. Pearson correlation between genes and metabolites was calculated, and the top-ranked correlations (| r| ≥ 0.9825 and *p* < 0.00046) were employed to construct the genes-metabolites co-expression network. The genes and metabolites were incorporated into their corresponding KEGG pathways.

An integrative analysis of the 69 DMs and 376 DEGs was undertaken using Pearson’s correlation. Then, a correlation-based (| r| ≥ 0.9825 and *p* < 0.00046) gene–metabolite co-expression network involving 35 DMs and 117 DEGs was constructed and visualized with Cytoscape (Supplementary Table 4 and Figure 6). We noted that amino acid metabolism-related pathways (glycine, serine, and threonine metabolism; aminoacyl-tRNA biosynthesis; and taurine and hypotaurine metabolism) and lipid metabolism-related signaling pathways (choline metabolism in cancer and sphingolipid signaling pathway) were highly represented in the network, as were several inflammation-related pathways (Toll-like receptor signaling pathway, NOD-like receptor signaling pathway, and chemokine signaling pathway) and neuron functions (neuroactive ligand-receptor interaction and ABC transporter). These results demonstrated that a well-defined regulatory network involving metabolite abundance, gene expression, and related pathways was altered in the hippocampus during the aging process.

## Discussion

Brain aging manifests as cognitive impairment and neurodegeneration, resulting in altered behaviors and neuronal function. In the present study, we adopted a multi-omics approach to examine the alterations in gene expression and metabolite abundance in the hippocampus of aged mice aiming to decipher the mechanisms underlying the aging process. The results showed that aged mice exhibited anxiety-like behaviors, decreased locomotor activity, and impaired associative learning and memory (Figures 1, 2). These findings are consistent with those of previous studies on aging-related behaviors (Shoji et al., 2016; Scott et al., 2017; Schroeder et al., 2021) and could be explained by changes in synaptic signaling resulting from alterations in gene and metabolite expression profiles (Table 1 and Figures 3–6).

Metabolomics or gene profiling is widely used to identify critical factors and pathways involved in the aging process. However, individually, these strategies do not provide the integrative information required for a more in-depth understanding of the changes that occur in the brain during aging. Multi-omics analysis represents an integrative strategy that combines transcriptomic, metabolomic, and phenotypic data and generates more comprehensive information relating to the molecular regulatory network underlying specific biological events. Previous multi-omics-related studies have identified several critical signaling pathways and mechanisms involved in the regulation of the aging process in several organisms, including increased mitochondrial stress; dysregulated redox, energy, and metabolic homeostasis; and epigenetic alterations (Wang et al., 2016; Hastings et al., 2019; Rivero-Segura et al., 2020; Adav and Wang, 2021; Gao et al., 2022). However, to date, no study has undertaken a multi-omics analysis of the aging hippocampus. Here, we first employed such a strategy (transcriptomics and non-targeting metabolomics) to decipher the molecular changes occurring in the aging hippocampus, and found that dysregulated amino acid and lipid metabolism and changes in MDM abundance collaboratively perturbed the hippocampus during the aging process (Table 1 and Figures 3, 5, 6), which corroborate previous studies using single omics analysis.

### Altered amino acid contents and dysregulated synapse functions

In this study, the greatest changes in the aged hippocampus were related to amino acid metabolic homeostasis, which provides supportive evidence for the recent aging review (Adav and Wang, 2021). Amino acids and peptides ccounted for ∼50% of the identified DMs in the aged hippocampus (Figure 3B), with most being downregulated (Figure 3C and Table 1). These DMs were enriched in the top-ranked KEGG pathways such as aminoacyl-tRNA biosynthesis; glycine, serine and threonine metabolism; and central carbon metabolism (Figures 3D, 6). Several of the DMs were either neurotransmitters or were involved in their synthesis. For instance, glycine is an inhibitory neurotransmitter that affects synaptic excitability and transmission and is involved in long-term synaptic plasticity-mediated learning and memory (Xu and Gong, 2010). Interestingly, the abundance of L-asparagine, the precursor for the neurotransmitter aspartate, and that of other amino acids such as L-serine, L-alanine, taurine, and glycine, was decreased in the aged hippocampus. The reduced levels of these amino acids likely disrupted neurotransmission, finally leading to dysregulated synaptic function, and this result was corroborated by the top-ranked pathways identified through RNA-seq analysis (Figure 5D). Of note, Trem2, was confirmed to upregulate in our study, and its upregulation would dysregulate synapse pruning, microglial activation, neuroinflammation, and synaptic function (Ulland and Colonna, 2018). Therefore, we speculated that the altered synaptic signaling contributes to the impairment of hippocampal synaptic plasticity (Bannerman et al., 2014) and might explain the learning and memory deficits and anxiety-like behaviors seen in our study (Figures 1, 2) and in other similar studies (Shoji et al., 2016; Scott et al., 2017; Schroeder et al., 2021).

In addition, we showed that the levels of histidine and several histidine-containing peptides (carnosine, homocarnosine, and homoanserine) were increased in the aged hippocampus, while histidine metabolism was found to be among the top-ranked KEGG pathways (Figures 3C,D, 6), which is consistent with previous similar studies (Ding et al., 2021). These peptides have been suggested to exert neuro-ameliorative effects; however, their defined functions and clinic therapeutic values are still debated (Petroff et al., 1998; Caruso et al., 2019; Schon et al., 2019). The abundances of betaine, creatine, and taurine were all significantly decreased. Betaine has been reported to have anti-inflammatory effects (Chen et al., 2021), while creatine and taurine are thought to provide energy to fuel the normal function of the brain (Chen et al., 2019; Roschel et al., 2021). Combined, these results suggested that the perturbation of amino acid metabolism may contribute to the aging of hippocampus through the modulation of synaptic plasticity, energy metabolism, and neuroinflammation.

### Decreased bioactive lipid metabolism accelerates the aging process in the hippocampus

As previous reports and reviews (Hamrick and Stranahan, 2020; Adav and Wang, 2021), lipids and lipid-like molecules constituted the second largest class of altered metabolites in the aged hippocampus in our data (Figures 3B,C, 4A and Table 1). Intriguingly, many of them were bioactive lipids known to exert protective functions in the brain. For instance, OEA, an endogenously produced metabolite, exerts neuroprotective effects by reducing neuroinflammation and improving spatial cognition (Mattace Raso et al., 2014). Meanwhile, PEA has anti-hyperalgesic and neuroprotective properties through modulating microglial activation, inflammation, and nociceptive pain (Mattace Raso et al., 2014; D’Aloia et al., 2021; Lama et al., 2021). However, OEA and PEA contents were significantly decreased in the aged hippocampus (Figure 4A), suggesting that their protective role was diminished with aging. In addition, OEA and PEA have also been implicated in the maintenance of gut barrier integrity and modulation of gut microbiota composition (Russo et al., 2018). Thus, supplementing these metabolites or improving their endogenous productions may improve cognitive and neuronal function and delay the aging process in the hippocampus.

### Gut microbiota-derived metabolites accelerate the aging process in the hippocampus

Gut microbiota-associated metabolism is essential for modulating metabolic profiles and is closely related to health in both humans and rodents (Agus et al., 2018; Wang et al., 2019). A Singaporean large cohort study showed the host–microbe–metabolite interplay during the aging process (Chen et al., 2022). Fecal transplantation from aged mice into young recipients has shown that the gut microbiota affects learning and memory by modulating hippocampal synaptic plasticity- and neurotransmission-associated proteins (D’Amato et al., 2020). Meanwhile, the same procedure also promoted brain neuroinflammation and an aging-like phenotype in young recipients (Parker et al., 2022). These observations indicate that a link exists between gut dysbiosis and brain age, which may aggravate brain aging through alterations in MDM contents (Rybnikova, 2018). Interestingly, we found that the abundance of several metabolites that are, or might be derived from, the gut microbiota was altered in the aged hippocampus. For example, TMAO, a well-defined MDM, was significantly increased in the aged hippocampus (Figures 3C, 4B), which is consistent with that previously reported (Scott et al., 2017; Ding et al., 2021). TMAO, initially reported as a critical risk factor for cardiovascular progression, was recently also demonstrated to affect cognition and promote the aging process (Bennett et al., 2013; Li et al., 2018; Govindarajulu et al., 2020; Brunt et al., 2021). Hypoxanthine, creatine, and hydroxyphenyllactic acid levels were decreased in the aged hippocampus (Figures 3C, 4B), which might also exacerbate the aging process (Biasibetti et al., 2017; Biasibetti-Brendler et al., 2018; Roschel et al., 2021). However, the slight increase in spermidine abundance in aged mice found in this study is not compatible with an aging phenotype, which indicates a possible metabolic feedback mechanism to antagonize aging. The functions of other differentially abundant MDMs identified in this study in the hippocampus and other brain regions remain unknown, which requires further investigation.

Besides those mentioned above, other identified DMs might also exert contrasting functions in the aging process (Figures 3C, 4C). For instance, low levels of thiamine (vitamin B1) can promote AD-like disorders, including neuritis plaques, tau hyperphosphorylation, and memory impairment (Gibson et al., 2016). Neu5Ac is known for its effects on brain development, cognition, and immune enhancement (Ling et al., 2022), while creatinine and hypoxanthine have been implicated in periphery energy dyshomeostasis (Harkness, 1988; Brosnan and Brosnan, 2007). These observations imply that changes in metabolite abundance may have a complex modulatory effect on the aging process in the hippocampus. It might find that the metabolite difference exists compared to the previous studies (Paban et al., 2010; Lin et al., 2016; Ding et al., 2021; Ge et al., 2021), which is possibly caused by variations in metabolomics approaches, sample preparations and the food and drink to the mice.

### Neuroinflammation activation in the aged hippocampus

Neuroinflammation is a leading cause of neurodegeneration, neurological disorders (Liang et al., 2017; Stuckey et al., 2021), and brain aging (Di Benedetto et al., 2017; Walker et al., 2022). Consistent with those in aging studies (Cribbs et al., 2012; Youm et al., 2013; Stilling et al., 2014; Di Benedetto et al., 2017; Mangold et al., 2017), our data indicated that the DEGs and DMs collectively affected the inflammation status in the aged hippocampus. For example, many of the downregulated DMs are known to exert anti-inflammatory effects. The levels of betaine, PEA, and OEA were decreased in the aged hippocampus (Figures 3C, 4A), indicative of diminished immunoprotection in this brain region. Meanwhile, TMAO is a neuroinflammation modulator that promotes inflammatory injury in the brain, affecting both neuronal and vascular integrity (Brunt et al., 2021; Lanz et al., 2022). These regulatory effects were further corroborated based on the inflammation-related pathways identified as being enriched in the RNA-seq analysis, such as microglial activation, interleukin-1β production, regulation of cytokines, and MAPK cascade (Figure 5C and Supplementary Figure 3). Several critical genes (*Trem2*, *Clec7a*, *Plau*, *Itgax*, and *Nlrp3*) were examined by reverse transcription and qPCR (RT-qPCR) (Figure 5I). Notably, *Trem2* was reported to be a risk gene in AD pathogenesis (Frank et al., 2008; Guerreiro et al., 2013) and its expression is upregulated in animal models of AD (Neumann and Takahashi, 2007; Guerreiro et al., 2013). The primary functions of TREM2 include the regulation of microglial activation and the maintenance of immune homeostasis (Neumann and Takahashi, 2007), and it is possible that it exerts similar effects in the aging hippocampus. The expression of the *Clec7a* and *Itgax* genes is upregulated in microglia and is associated with neurodegenerative progression (Hunter et al., 2021). NLRP3, a component of the NLRP3-inflammasome, is also a mediator of neuroinflammation and its dysregulation has been implicated in neurodegeneration (Ising et al., 2019; Pellegrini et al., 2020). *Plau* is a gene related to aging and age-related diseases (Cardoso et al., 2018) and has been suggested to exert chronic inflammatory effects (Cardoso et al., 2018; Dowsett et al., 2021). Our integrated pathway analysis showed that neuroinflammation-related pathways (Walker et al., 2022) such as Toll-like receptor signaling pathway, NOD-like receptor signaling pathway, cytokine-cytokine receptor interaction, NF-kB signaling pathways, JAK-STAT signaling pathway, PI3K-Akt signaling pathway, and Sphingolipid signaling pathway were the top-ranked KEGG pathways. These findings were consistent with those of other aging-related studies (Liang et al., 2017; Walker et al., 2022) and implied that neuroinflammation-related pathways are activated in the aged hippocampus. Furthermore, our metabolic profiling identified and highlighted several critical regulators in neuroinflammation, reinforcing the neuroinflammation concept in previous studies (Youm et al., 2013; Pardo et al., 2017).

As shown in previous studies (Cardoso et al., 2018), cell death-related pathways, hippocampal neuron apoptotic process, and positive regulation of cell death were also among the top-ranked pathways (Figure 5E). Moreover, a pool of genes was clustered in these pathways, and several of these genes (*S100a8*, *Trem2*, and *Clec7a*) were validated by RT-qPCR (Figure 5I), indicating that cell injury was present in the aged hippocampus. Our gene-pathway analysis showed the tissue homeostasis-related pathways (phagocytosis, endopeptidase activity, hydrolase activity, calcium ion homeostasis, and superoxide anion generation) (Figures 5C,F–H and Supplementary Figure 3I) were the top-ranked as previously described (Hamezah et al., 2018). Therefore, we speculate that tissue dyshomeostasis accounts for the cell injury of the aged hippocampus.

In addition, we construct the co-expression work that integrates several neuroinflammation pathways and metabolism in the aging hippocampus, which is not well defined (Lanke et al., 2018). Although an integrated multi-omics analysis was applied in the present study, the identified metabolites and DMs were, at some degree, different from previous ones, as caused by the variations of sensitivity and precision of the different metabolomics approaches. In addition, the small sample seize and only male mice discussed here is another weakness of this study. Therefore, much work based on a more cohort and comprehensive omics level needs further studies.

## Conclusion and prospects

In conclusion, we firstly employed a multi-omics approach to comprehensively analyze the metabolites, genes, and related signaling pathways that are altered in the hippocampus during the aging process. We identified 69 DMs and 376 DEGs in the aged hippocampus. Among them, 35 metabolites and 119 potential target genes, constituting the top 200 correlations, were employed in a co-expression network integrating pathways enriched with DEGs and DMs. Furthermore, the identified DMs and DEGs were found to be involved in several metabolism-related pathways, including amino acid metabolism, lipid metabolism, neuroinflammation-related pathways, synapse function, cell death and the maintenance of cellular/tissue homeostasis. Importantly, our data hinted that altered MDMs might mediate the interaction between the gut and brain during aging. Collectively, we have generated comprehensive omics data that provides an in-depth understanding of the molecular changes occurring in the hippocampus during the aging process.

The gut-brain axis theory pioneer the brain’s function regulation in a fantastic way, but the inter-crosstalk between the gut and brain remains unknown. The present research showed MDMs might be the mediator in bridging the gut and brain. Several MDMs were identified in our work, which provides evidence of their interaction between gut and brain, i.e., gut-brain axis functions in the aging process. However, the specific role of the mentioned MDMs in the aging process needs further investigation, including their transportation, metabolism, functions and gender difference *in vivo*.

## Data availability statement

The raw reads of RNA-seq data have been deposited in the NCBI sequence read archive (SRA) and assigned a BioProject accession number (PRJNA842200) for access. In addition, other raw data could be made available on reasonable request to the corresponding authors (YL, yinzhonglu@shsmu.edu.cn or JZ, Junjie.zhang@shsmu.edu.cn).

## Ethics statement

This animal study was reviewed and approved by Ethics committee of Tongren Hospital, Shanghai Jiao Tong University School of Medicine.

## Author contributions

YL and JZ designed the experiments. YL, KX, and XD performed the experiments. YL, DL, SW, RF, XD, and JZ analyzed the data. JZ supervised the study. YL, GC, and JZ wrote the manuscript. All authors reviewed the results and approved the final version of the manuscript.

## Footnotes

1
http://www.genome.jp/kegg/

2
https://cloud.oebiotech.cn/task/

3
https://www.ncbi.nlm.nih.gov/assembly/GCF_000001635.22/

4
http://ccb.jhu.edu/software/hisat2/index.shtml

5
https://cloud.oebiotech.cn

6
https://www.ncbi.nlm.nih.gov/bioproject/

## Supplementary material

Supplementary Figure 1 Non-targeted metabolomics analysis of hippocampus tissue from young and aged mice.

Supplementary Figure 2 PCA and volcano analysis of RNA-seq analysis of the genes in the ageing hippocampus.

Supplementary Figure 3 Hierarchical heatmap analysis of the differential gene expression.

Supplementary Table 1 List of metabolites identified in aged and young hippocampal tissue of mice.

Supplementary Table 2 List of microbiota-derived metabolites identified in aged hippocampus.

Supplementary Table 3 List of differentially expressed genes.

Supplementary Table 4 Top200 correlations of differential mRNA-differential metabolite.
