# Supplementary material for: Multi-omics analysis reveals neuroinflammation, activated glial signaling, and dysregulated synaptic signaling and metabolism in the hippocampus of aged mice
Source: Front Aging Neurosci. 2022 Nov 3;14:964429. doi: 10.3389/fnagi.2022.964429 (PMC9669972; doi:10.3389/fnagi.2022.964429)
Supplement: Supplementary Figure 1 — Non-targeted metabolomics analysis of hippocampus tissue from young and aged mice. [file Image_1.PDF]

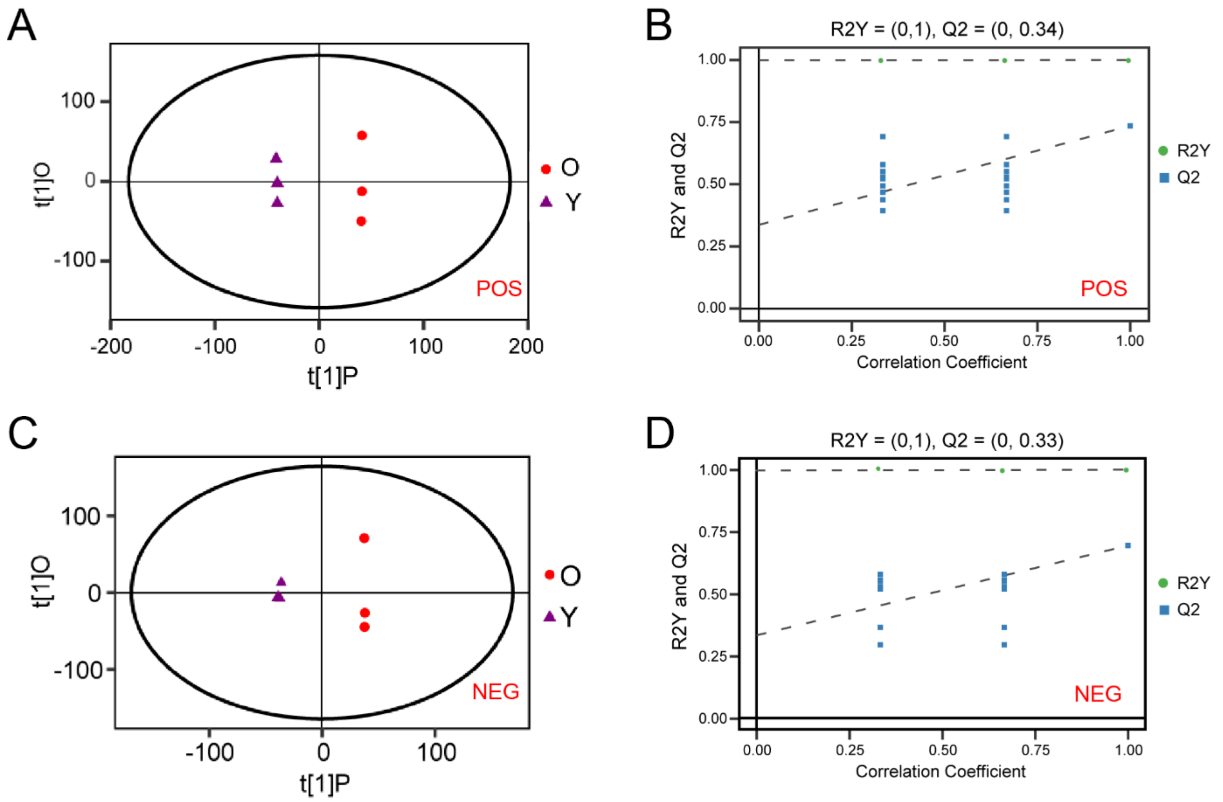

Supplementary Figure 1. Non-targeted metabolomics analysis of hippocampus tissue from young and aged mice. OPLS-DA analysis of the whole metabolites by LC/MS-based metabolomics detection (**A, C**) and permutation tests of the metabolites in POS or NEG ion detection model (**B, D**).

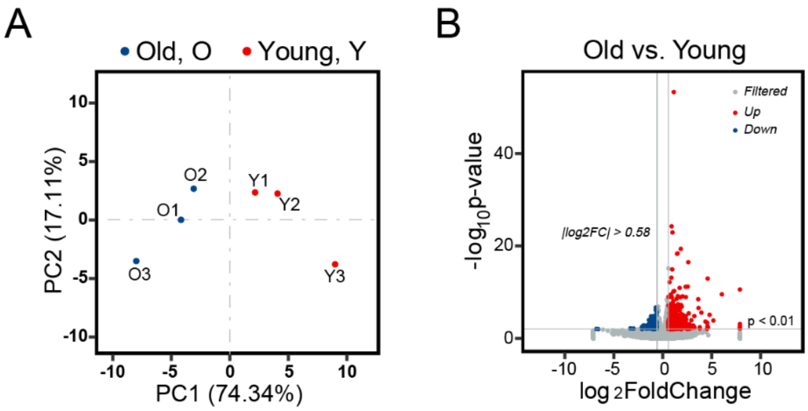

Supplementary Figure 2. PCA and volcano analysis of RNA-seq analysis of the genes in the ageing hippocampus.

(**A**)The Principal Component Analysis (PCA) of whole genes; (**B**)Volcano map of whole differential expression genes (DEGs).

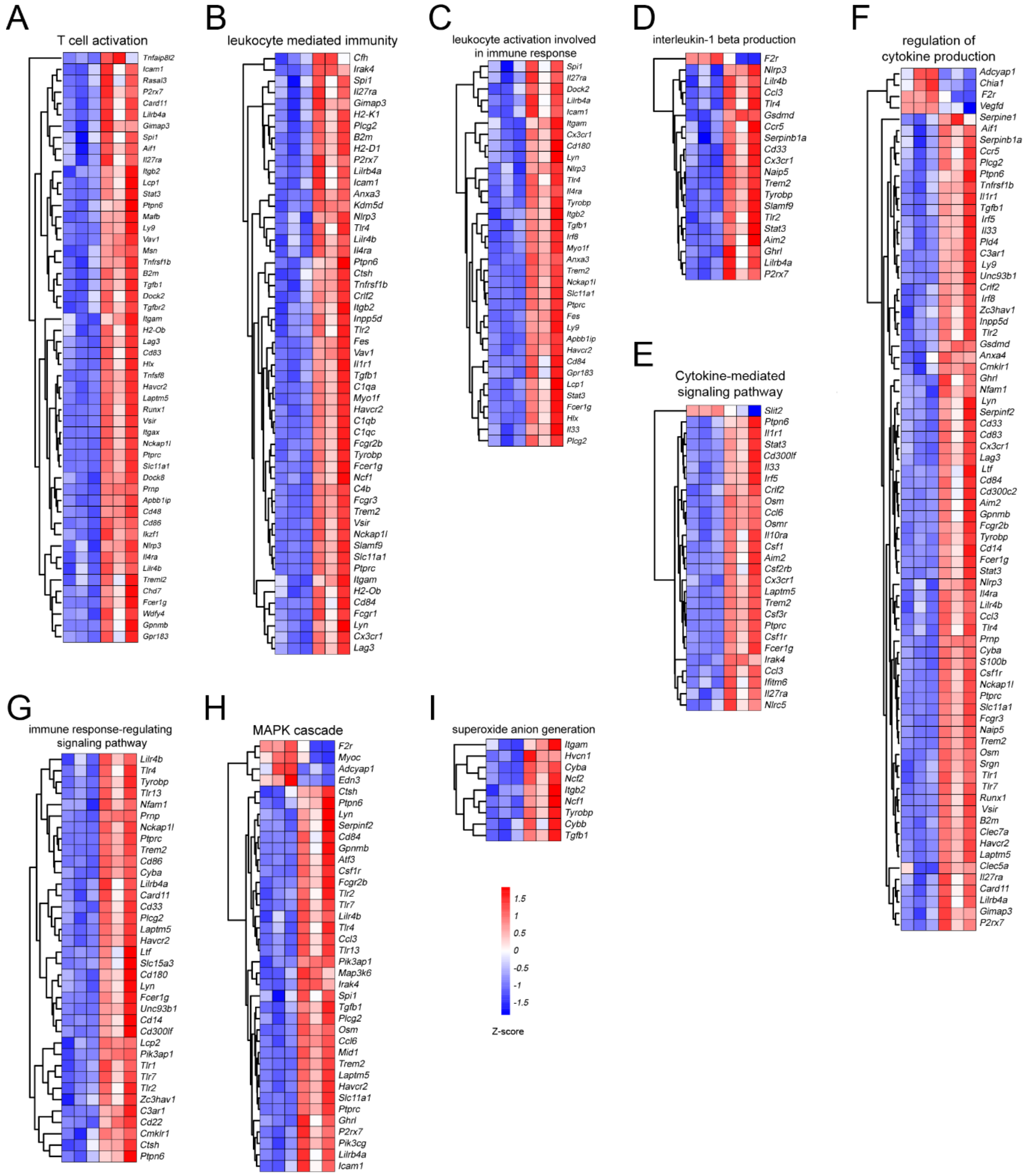

Supplementary Figure 3. Hierarchical heatmap analysis of the differential gene expression. The differential gene expression showed in GO term enriched in the hippocampus of aged mice.
